# Ferroptosis, necroptosis, and pyroptosis in the occurrence and development of ovarian cancer

**DOI:** 10.3389/fimmu.2022.920059

**Published:** 2022-07-25

**Authors:** Chunmei Zhang, Ning Liu

**Affiliations:** Department of Obstetrics and Gynecology, Shengjing Hospital of China Medical University, Shenyang, China

**Keywords:** ovarian cancer, ferroptosis, necroptosis, pyroptosis, malignant progression

## Abstract

Ovarian cancer (OC) is one of the most common malignancies that causes death in women and is a heterogeneous disease with complex molecular and genetic changes. Because of the relatively high recurrence rate of OC, it is crucial to understand the associated mechanisms of drug resistance and to discover potential target for rational targeted therapy. Cell death is a genetically determined process. Active and orderly cell death is prevalent during the development of living organisms and plays a critical role in regulating life homeostasis. Ferroptosis, a novel type of cell death discovered in recent years, is distinct from apoptosis and necrosis and is mainly caused by the imbalance between the production and degradation of intracellular lipid reactive oxygen species triggered by increased iron content. Necroptosis is a regulated non-cysteine protease–dependent programmed cell necrosis, morphologically exhibiting the same features as necrosis and occurring *via* a unique mechanism of programmed cell death different from the apoptotic signaling pathway. Pyroptosis is a form of programmed cell death that is characterized by the formation of membrane pores and subsequent cell lysis as well as release of pro-inflammatory cell contents mediated by the abscisin family. Studies have shown that ferroptosis, necroptosis, and pyroptosis are involved in the development and progression of a variety of diseases, including tumors. In this review, we summarized the recent advances in ferroptosis, necroptosis, and pyroptosis in the occurrence, development, and therapeutic potential of OC.

## Introduction

Ovarian cancer (OC) is one kind of gynecologic malignancies with high mortality (1) and annually increased incidence (2, 3), which seriously threatens women’s life and health. OC is prevalent in middle-aged and elderly women. There is often no obvious clinical manifestation in the early stage. About 70% of patients first present with abdominal distention, ascites, and abdominal pain (4). Therefore, more than 75% of patients with OC present with advanced stages at the time of first or confirmed diagnosis (5, 6). In clinical settings, OC is featured with insidious onset, lack of early diagnostic markers, high malignancy, easy metastasis, and poor prognosis (7, 8). Currently, surgery combined with platinum and paclitaxel-based chemotherapy is the mainstream treatment for OC (7, 9). Although surgery and chemotherapy have significantly improved the prognosis of patients with OC in recent years, the 5-year survival rate of patients with advanced OC remains low (10, 11) because most patients with OC are advanced at the time of diagnosis and some patients with OC develop chemoresistance later following treatment (12, 13). Therefore, the search for potential biomarkers and therapeutic targets is of great clinical importance for early screening of patients with OC and improving the prognosis of patients with OC.

Cell death is a life phenomenon and an irreversible life process of cells. Cell death plays an indispensable role in the biological process of maintaining the normative homeostasis of the body and inhibiting the rapid proliferation of tumor cells (14, 15). Cell death includes regulated cell death (RCD) and accidental cell death (ACD) (16–18). RCD is a genetically determined form of active and ordered cell death that plays an important role in the maintenance of homeostasis (19, 20). Currently, the common types of RCD include apoptosis, necroptosis, ferroptosis, autophagy, and pyroptosis (21). Ferroptosis, a newly discovered non-apoptotic form of cell death, is essentially iron ion–dependent RCD. Necroptosis is mainly mediated by cytokines (TNF-α, IFN-α, and IFN-γ), Toll-like receptors (TLR3, TLR4, and TLR9), and nucleic acid (DNA and RNA) receptors. RIPK1/3 and MLKL are involved in the development of necroptosis, with MLKL being the key molecule (22, 23). Pyroptosis, which is a pathological form of suicide of cells distinct from apoptosis, is mainly mediated by Caspase-1 and Caspase-4/5/11. Pyroptotic signaling pathway mainly includes classical pyroptosis pathway and non-classical pyroptosis pathway, with inflammatory vesicle production and Gasdermin D (GSDMD) activation as the hallmarks of pyroptosis pathway (24, 25). Gasdermin B (GSDMB) is highly expressed in inflammatory bowel disease and contributes to the progression of inflammation by disrupting epithelial barrier function and promoting the development of ferroptosis (26). Necroptosis is mainly mediated by cytokines (TNF-α, IFN-α, and IFN-γ), Toll-like receptors (TLR3, TLR4, and TLR9), and nucleic acid (DNA and RNA) receptors. RIPK1/3 and MLKL are involved in the development of necroptosis, with MLKL being the key molecule (22, 23). Necroptosis is induced by cigarette smoke exposure and is increased in the lungs of patients with chronic obstructive pulmonary disease (COPD) and patients with experimental COPD. Inhibition of necroptosis attenuated cigarette smoke-induced airway inflammation, airway remodeling, and emphysema (27). Recently, accumulating evidence has showed that ferroptosis, necroptosis, and pyroptosis play an important role in tumor development. Expression levels of ZBP1 are significantly increased in necrotic tumors. In addition, ZBP1 deficiency blocked necroptosis and significantly inhibited tumor metastasis during breast cancer development (28). In breast cancer, DRD2 promotes M1 polarization of macrophages and triggers GSDME-executed pyroptosis that regulates the tumor microenvironment and inhibits tumor malignant progression (29). All these pieces of evidence highlight the important roles of ferroptosis, necroptosis, and pyroptosis in the progression and metastasis of human malignancies.

RCD in malignancies has been extensively studied and more and more pieces of evidence reveals that ferroptosis, necroptosis, and pyroptosis are highly involved the development, progression, and regression of OC (30, 31). In this paper, we reviewed the molecular mechanisms of ferroptosis, necroptosis, and pyroptosis and their regulatory roles in OC, providing a new perspective on the pathogenesis and targeted therapy of OC and exploring their potential as potential therapeutic targets for death.

## Ferroptosis

### The overview of ferroptosis

Programmed cell death plays an important biological effect in maintaining the homeostasis of the organism. As a novel mode of cell death, ferroptosis was first described in 2012. The small-molecule inhibitor erastin was found to induce a unique mode of cell death in ras mutant cells that could not be rescued by apoptosis inhibitors and necrosis inhibitors but was reversed by the iron ion chelator deferoxamine. Later, this novel mode of cell death was named ferroptosis (32). Ferroptosis is an iron- and ROS-dependent mode of cell death, which is characterized by major cytological changes including reduction or loss of mitochondrial cristae, rupture of the outer mitochondrial membrane, and mitochondrial membrane ruffling (33, 34). All these above changes are caused to loss of selective permeability of the cell membrane due to the occurrence of peroxidation of lipid components of the cell membrane and oxidative stress (35, 36). In addition, different physiological conditions and pathological stresses have been found to induce tissue ferroptosis (37, 38). Ferroptosis is gradually recognized as an adaptive process by which the body eliminates malignant cells by removing cells damaged by nutritional deficiency, infection, or stress (39–41). Thus, ferroptosis has an inhibitory effect on tumorigenesis under normal conditions, and abnormalities in the oxidative stress pathway are an important cause of ferroptosis. Although tumor cells are in a constant state of excessive oxidative stress, they are less likely to develop ferroptosis, which is mainly dependent on their own antioxidant system (42, 43). In-depth studies based on the mechanisms of ferroptosis occurrence and regulation in tumor cells are of great clinical importance for the formation of new strategies for tumor therapy.

### Regulatory mechanisms of ferroptosis

**Figure 1:**
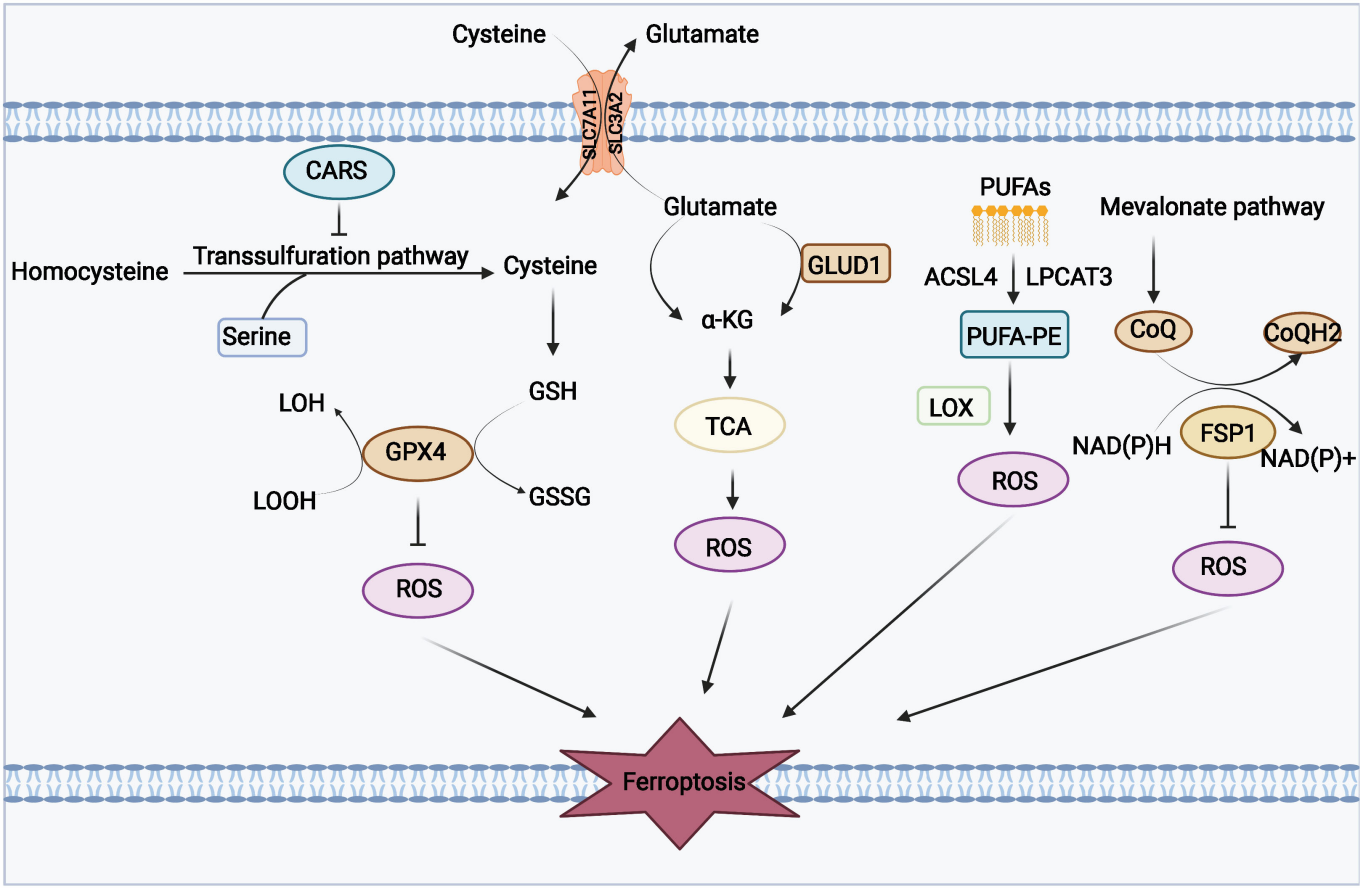
Mechanisms of amino acids and lipid metabolism in ferroptosis. Cysteine can be transported into the cell, whereas glutamate can be transported out of the cell by the Xc-system. Cysteine can be used to synthesize glutathione to maintain the balance of the redox state, and it can also be synthesized through the transsulfurization pathway blocked by CARS. Glutamate can be converted to a-KG by transaminase or GLUD1 pathway and participate in TCA, thereby generating ROS. PUFAs derived from cell membranes can be catalyzed by ACSL4 and LPCAT to PUFA-PE, and PUFA-PE can be peroxidized by the LOX family. FSP1 and coenzyme Q also play an important role in the antioxidant system of coenzyme Q.

**Figure 2:**
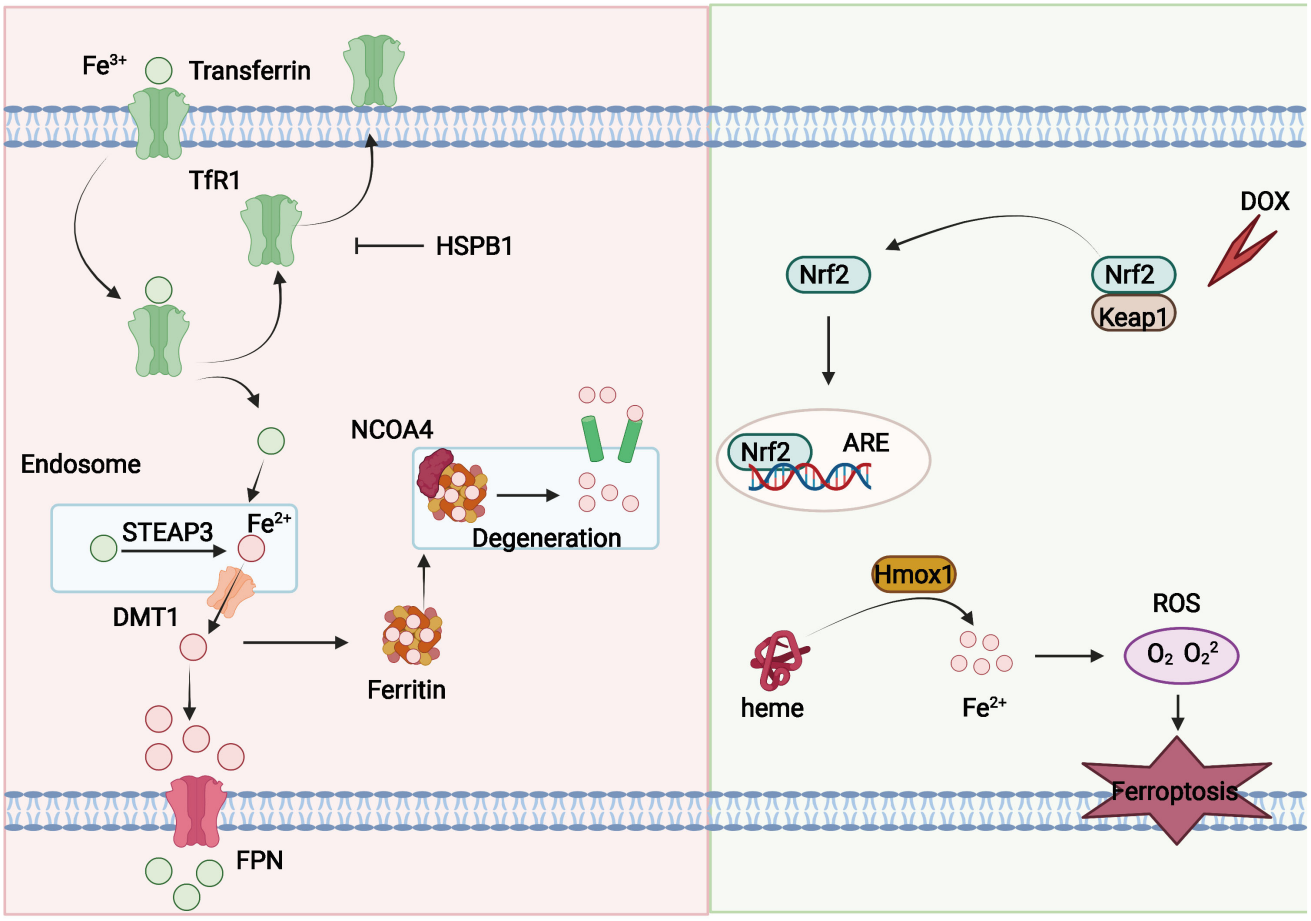
Mechanisms of iron metabolism in ferroptosis. Fe^3+^ can couple to transferrin and enter the intercellular milieu mediated by TfR1. Transferrin can be recycled and exported extracellularly and blocked by HSPB1. Fe^3+^ is reduced to Fe^2+^ by DMT1 in endosomes, and Fe^2+^ can be transported into the cytoplasm. Fe^2+^ can be released from ferritin through NCOA4-mediated ferritin phagocytosis, and part of Fe^2+^ can be exported outside the cell and oxidized by FPN. In addition, DOX can also induce ferroptosis. Cardiac output of DOX activates the Keap1/Nrf2 pathway, and Nrf2 further activates the downstream protein Hmox1 and prompts it to oxidize heme and release iron, leading to ferroptosis.

Phospholipid hyperoxidation of polyunsaturated fatty acids in the cytoplasmic membrane has been shown to be the most important driver of ferroptosis (44). The proportion of polyunsaturated fatty acids in lipids determines the ease with which lipid peroxidation occurs in cells. Acyl-coa synthetase long-chain family member 4 (ACSL4) and lysophosphatidylcholine acyltransferase 3 (LPCAT3) are key enzymes that regulate polyunsaturated fatty acid synthesis in phospholipid membranes, whereas the inhibition of both ACSL4 and LPCAT3 promotes ferroptosis resistance (45). There are two types of intracellular lipid peroxidation, namely, non-enzymatic and enzymatic lipid peroxidation. Non-enzymatic lipid peroxidation, also known as lipid autoxidation, is mainly a free radical-mediated chain reaction in which hydroxyl radicals generated by the Fenton reaction oxidize polyunsaturated fatty acids to lipid hydroperoxides (46). In contrast, enzymatic lipid peroxidation is mainly a process of direct oxidation of free polyunsaturated fatty acids to various types of lipid hydroperoxides catalyzed by lipoxygenase (LOX) (47, 48). Lipid hydroperoxides are catalyzed by iron ions to generate alkoxy radicals, which participate in the next lipid peroxidation chain reaction, ultimately leading to cell death (49). The exact mechanism by which lipid peroxidation leads to cellular ferroptosis is still not well understood. It could be due to the formation of structural lipid gaps, similar to protein gaps in necrosis and focal death. It is also possible that the depletion of polyunsaturated fatty acids causes structural changes in the fluidity of the cell membrane as well as an increase in membrane permeability, ultimately leading to loss of membrane integrity (50). In addition, lipid peroxides can be broken down into toxic aldehydes, which can further enhance the lipid peroxidation of ferroptosis by promoting protein inactivation through cross-linking reactions (51, 52). The mechanisms of amino acids and lipid metabolism in ferroptosis were displayed in **Figure 1**. Iron ions are important catalysts for lipid peroxidation reactions. Intracellular uptake, release, and storage of iron ions are all important regulators of ferroptosis. Inhibition of nitrogen fixation 1 (NFS1), which provides sulfur from cysteine for the synthesis of iron-sulfur clusters, activates the iron starvation response by simultaneously increasing transferrin receptor (TFRC) expression and degrading ferritin heavy chain 1 (FTH1), causing an increase in free iron ions, thereby making cells sensitive to ferroptosis activator (53–55). Overactivated heme oxygenase 1 increases intracellular free iron content and enhances ferroptosis effect by degrading hemoglobin into free iron, biliverdin, and carbon monoxide (56–58). Nuclear receptor coactivator 4 (NCOA4) recognizes intracellular after ferritin recognition, and ferritin transfers stored ferric ions to the autophagosome for degradation, which, in turn, releases ferric ions into the cytoplasm to become free iron, a process also known as iron autophagy (59, 60). In addition, genes such as nuclear receptor coactivator (NRF2) and heat shock protein B1 have been found to affect the sensitivity of cells to ferroptosis inducers by regulating intracellular iron ion metabolism (61, 62). This shows that iron ion metabolism is a potential regulatory point for the induction of cellular ferroptosis. The mechanisms of iron metabolism in ferroptosis were displayed in **Figure 2**. In addition to iron metabolism and lipid peroxidation responses, a wide range of intracellular antioxidant mechanisms also plays an important role in regulating ferroptosis sensitivity (63, 64). Glutathione (GSH), the most important intracellular antioxidant metabolite, requires cysteine as a raw material for its synthesis, and cells can endogenously synthesize cysteine *via* the transsulfuration pathway to resist ferroptosis (65, 66). In addition, myristoylation modification of ferroptosis inhibitory protein 1 leads to a nicotinamide adenine dinucleotide (NADH)–dependent decrease in coenzyme Q, which acts as a radical-trapping antioxidant to inhibit lipid peroxide proliferation (67, 68).

## Necroptosis

### The overview of necroptosis

Necroptosis, also known as programmed necrosis, is a necrosis-like form of cell death that relies on receptor interacting serine/threonine protein kinase 1 (RIPK1), RIPK3, and mixed lineage kinase domain–like pseudokinase (MLKL) and does not depend on cysteine-aspartic proteases 1 (caspase-1) (23, 69, 70). Necrosis was once thought to be passive and non-programmed, but recent studies have revealed that cell necrosis is an active and modifiable process (71). In the absence or inhibition of caspase-8 or Fas-associating protein with a novel death domain (FADD), cells induced by tumor necrosis factor α (TNF-α) still die, and the cell death morphology resembles that of necrotic cells (72–74), gradually revealing a caspase-independent cell death similar to necrosis. Degterev et al. first described the role of the small-molecule Nec-1 in regulating cell necrosis, updating, for the first time, the concept of unregulated necrosis to cells that can be regulated by Nec-1 necroptosis (16, 75). Since then, necroptosis has been defined as programmed necrosis, whereby cells undergoing necroptosis have their cell membranes ruptured, releasing intracellular material that can stimulate a variety of cells (including macrophages, fibroblasts, and endothelial cells) to participate in the intrinsic immune response and exacerbate the inflammatory response by releasing inflammatory cytokines, leading to a dual role for necroptosis in different physiological or pathological processes (22, 23).

### Regulatory mechanisms of necroptosis

**Figure 3:**
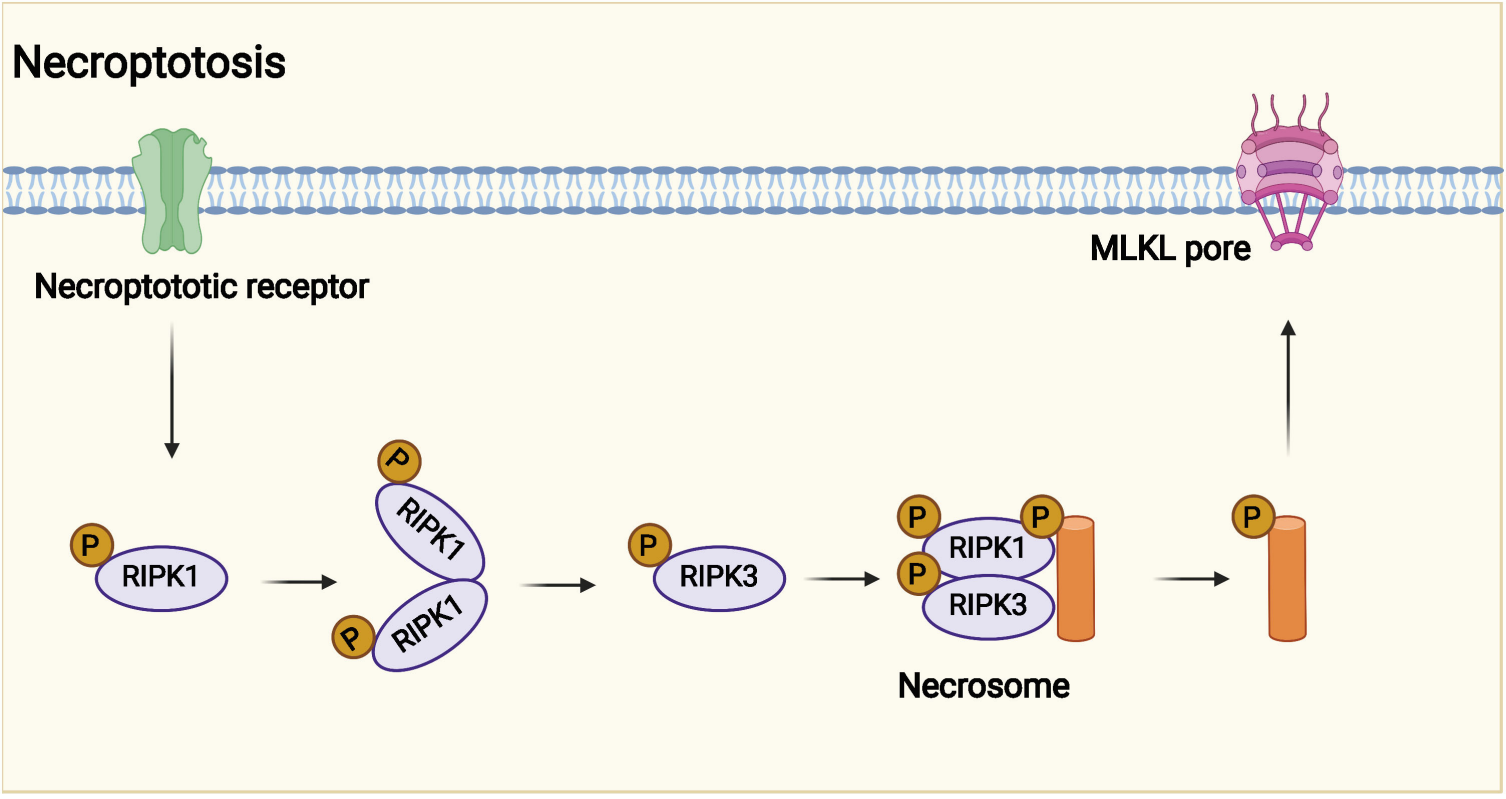
Potential mechanism of necroptosis. Necroptotic death may have evolved into the innate immune mechanism that complements apoptosis to eliminate pathogens. Necroptosis is affected by receptor interacting protein kinase 3 (RIPK3) and mixed lineage kinase domain-like protein (MLKL).

The potential mechanism of necroptosis is shown in **Figure 3**. The programmed cell death pattern is driven by RIPK1 through its kinase function, including through the formation of complex iia leading to apoptosis and complex iib leading to necroptosis (18, 76, 77). After TNF-α interacts with TNFR, TNFR1 starts to recruit the downstream protein molecules TNFR1- associated death domain protein (TRADD), RIPK1, TRAF2/5, and linear ubiquitin chain assembly complex (LU⁃BAC) proteins to form complex I, in which RIPK1 is polyubiquitinated and activates nuclear RIPK1 polyubiquitinates and activates the nuclear factor κB (NF-κB) and mitogen-activated protein kinase (MAPK) signaling pathways, inhibiting caspase-8 activation and promoting cell survival (72, 78–80). If TNF-α recruits TRADD, FADD, pro–caspase-8, and RIPK1 to form complex iia, then complex iia promotes activation of caspase-8, and activated caspase-8 undergoes apoptosis by activating caspase-3 (73, 81, 82). When caspase-8 is inhibited or its activity level is relatively low, RIPK1 recruits RIPK3 and both recruit MLKL through the RIP homotypic interaction motif (RHIM) to form complex iib, also known as necrosome (83, 84). MLKL phosphorylation causes oligomerization and membrane localization. Oligomerized MLKL has the ability to bind directly to lipids, allowing polymerized MLKL to form membrane permeable pores, disrupt cell membrane integrity, and undergo necroptosis (71, 85). However, it inhibits the formation of complex iib to inhibit necroptosis, so the role of RIPK1 in cells can be determined by targeting the drug to determine whether the cells survive or undergo necroptosis (74, 86, 87).

## Pyroptosis

### The overview of pyroptosis

Pyroptosis is a caspase-1–mediated mode of programmed cell death (88, 89) characterized by rapid plasma membrane rupture followed by the release of cellular contents and pro-inflammatory substances such as IL, which triggers an inflammatory cascade response that results in cellular damage (24, 90). As early as 1992, Zychlinsky et al. observed experimentally that Shigella fowleri could induce lytic death in infected host macrophages (91). In 2001, Cookson et al. showed that this form of death was caspase-1 activity–dependent, unlike caspase-3 activity–dependent apoptosis (92). They first defined focal cell death as a caspase-1–dependent form of cell death. Furthermore, Shao et al. showed that cell scorch death can also be induced by the activation of caspase-4/5/11 by intracytoplasmic LPS and that activated caspase-4/5/11 ultimately induces cell scorch death through cleavage of Gasdermin family proteins. Therefore, they defined cell scorch death as programmed cell necrosis mediated by the Gasdermin family (93, 94). When cell death occurs, the nucleus is condensed, chromatin DNA is randomly broken and degraded, numerous pores appear in the cell membrane, the cell membrane loses its ability to regulate the entry and exit of substances, the cell loses internal and external ionic balance, osmotic swelling occurs, and the membrane ruptures, releasing cell contents and other active substances, stimulating the body’s immune response, and recruiting more inflammatory cells. This stimulates the body’s immune response, recruits more inflammatory cells, and amplifies the inflammatory response.

### Regulatory mechanisms of pyroptosis

**Figure 4:**
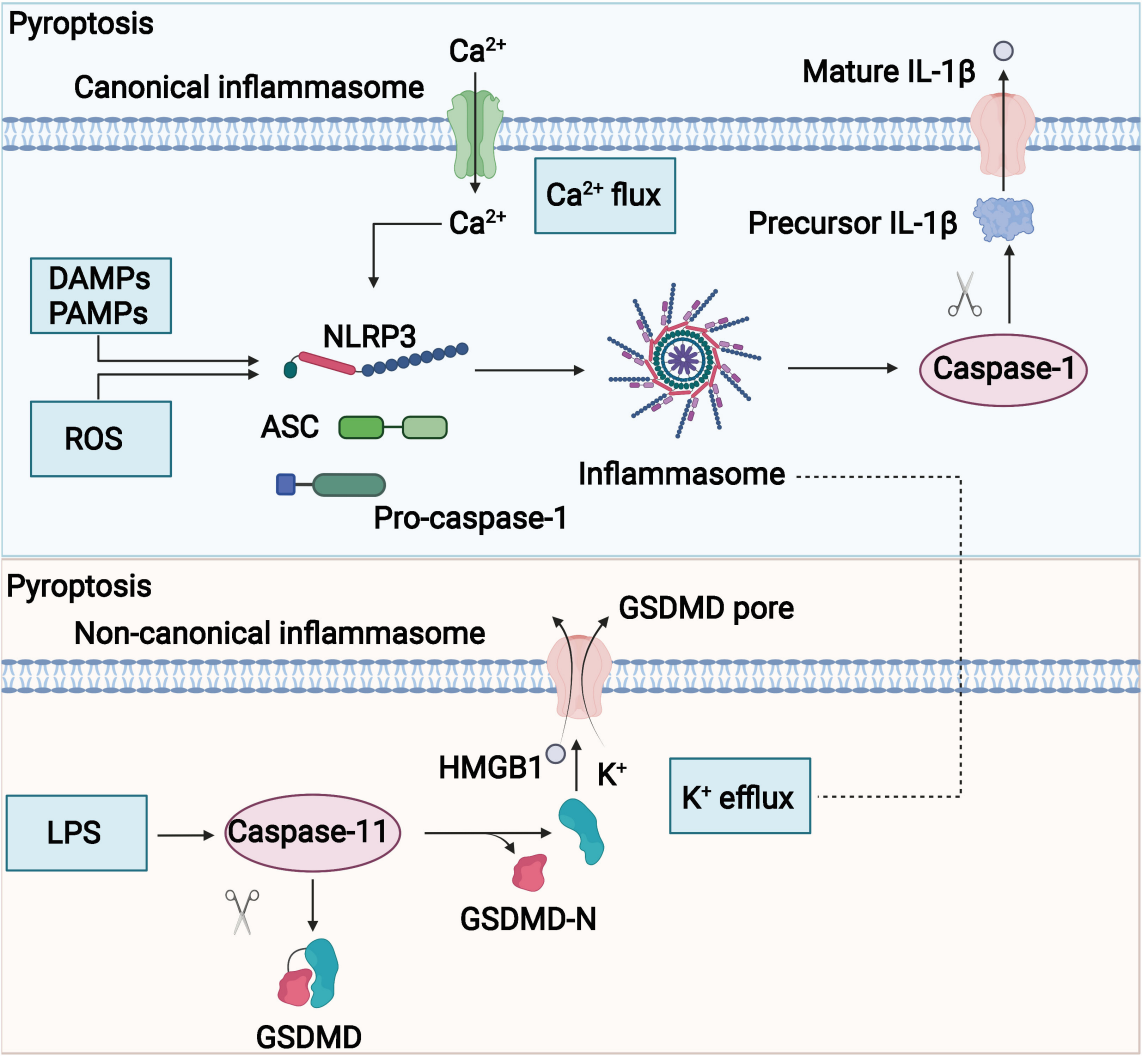
Potential mechanism of pyroptosis. The molecular mechanisms of pyrolysis mainly include canonical and noncanonical signaling.

The potential mechanism of pyroptosis is shown in **Figure 4**. The assembly of the inflammasome is the initiating step of the classical pathway of pyroptosis, a complex of intracellular macromolecular proteins necessary for inflammation to occur and capable of recognizing dangerous signaling molecules such as bacteria and viruses. The inflammasome is mainly composed of pattern recognition receptors (prrs), apoptosis-associated speck-like protein (ASC), and pro–caspase-1 (95–98). Among them, prrs are receptor proteins responsible for the recognition of different intracellular signaling stimuli, which are mainly composed of nucleotide-binding oligomerization domain–like receptor protein 1 (NLRP1) and NLRP3; nucleotide ASC is an articulated protein that consists mainly of the N-terminal pyrin domain (PYD), caspase activation, and recruitment domain (CARD) (95, 99). Pro–caspase-1 is an effector molecule that is activated to specifically cleave GSDMD. Danger-signaling sensors after the recognition of the danger signaling molecule by NLRP1, NLRP3, or AIM2 bind to the PYD structural domain at the N-terminal end of the bridging protein through its N-terminal PYD structural domain, and ASC then recruits caspase-1 through the interaction of the CARDCARD structural domain to complete the assembly of the inflammasome (100, 101). The inflammasome acts by activating the effector molecule pro–caspase-1 to form active caspase-1. Activated caspase-1 is able to specifically cleave the GSDMD to generate the N-terminal and C-terminal ends, and the N-terminal end of the GSDMD binds to cell membrane phospholipids, causing many small pores to form in the cell membrane (102, 103). The integrity of the cell is disrupted, and the water flows inward, leading to cell swelling and rupture, releasing intracellular inflammatory substances, and inducing pyroptosis (104). In addition, caspase-1 also promotes the maturation of IL-18 and IL-1β precursors, which are cleaved into active IL-18 and IL-1β (90, 105, 106) and secreted through the cell membrane pores to the outside of the cell, recruiting more inflammatory cells and causing an inflammatory waterfall response. This caspase-1–mediated cell death is called the classical pathway of pyroptosis. The non-classical pathway of pyroptosis is mainly mediated by caspase-4, caspase-5, and caspase-11. Upon stimulation of cells by bacterial LPS, caspase-4, caspase-5, and caspase-11 bind directly to and are activated by bacterial LPS (40, 107). Activated caspase-4, caspase-5, and caspase-11 specifically cleave the GSDMD, relieving intramolecular inhibition of the GSDMD N structural domain (108), and the GSDMD N terminus binds to cell membrane phospholipids, causing cell membrane pore formation, cell swelling, rupture, and induction of pyroptosis (108). The GSDMD N terminus also amplifies the inflammatory response by activating the NLRP3 inflammasome, which, in turn, activates caspase-1 (109), which stimulates the maturation of IL-18 and IL-1β precursors and secretes IL-18 and IL-1β extracellularly (110).

## Role of ferroptosis, necroptosis, and pyroptosis in tumors

Ferroptosis is a novel form of RCD induced by iron-dependent lipid peroxidation damage, which is morphologically, genetically, and molecularly different from other cell death modalities such as apoptosis, autophagy, and necrosis. The relationship between ferroptosis and tumors is extremely close, and there are numerous studies to design and develop ferroptosis-based anticancer drugs, and ferroptosis is expected to be a novel therapeutic approach for tumors. The interactive dialogue between triple-negative breast cancer (TNBC) cells and tumor-associated macrophages (tams) promotes the sustained activation of HLF in tumor cells through the IL-6–TGF-β1 axis. Subsequently, hepatic leukemia factor (HLF) promotes resistance to ferroptosis in TNBC cells *via* GGT1, ultimately promoting malignant tumor progression (1). The current design of corresponding compounds targeting key molecules in ferroptosis can effectively inhibit tumor progression with significant clinical translational implications. Mimetic drugs composed of small-molecule inducers of ferroptosis, erastin, and RSL3 with BH3 were effective in synergistically killing U251 cells and inhibiting malignant progression of glioblastoma (111). Nanocatalytic activity leads to simultaneous inhibition of GPX4/GSH and FSP1/coq10h2 pathways and synergizes with the GPX4 inactivation function of RSL3 to cause significant ferroptosis damage and thus inhibit malignant progression of triple-negative breast cancer (112). Depletion of PSTK leads to inactivation of GSH peroxide 4 (GPX4) and inhibition of selenocysteine and cysteine synthesis. GSH metabolism is disrupted due to inhibition of selenocysteine and cysteine synthesis, which enhances the induction of ferroptosis after targeted chemotherapy, leading to malignant progression of hepatocellular liver cancer (113). The use of PSTK inhibitor-punicalin together with sorafenib for the treatment of HCC *in vitro* and *in vivo* exhibits synergistic effects.

Research on targeted tumor therapy based on necroptosis is currently underway, suggesting that necroptosis will provide a new strategy for tumor treatment. Disintegrin and Metalloprotease 17 (ADAM17) was identified as a novel important regulator of necroptosis whose activity could significantly affect the role of TNFR1-dependent tumor cell induction of endothelial cell death, tumor cell extravasation, and subsequent metastatic seeding (114). Furthermore, mediated TNFR1 extracellular domain shedding and subsequent processing by the γ-secretase complex are key enzymatic steps in the induction of TNF-induced necrotic apoptosis.ADAM17 may serve as an important target as an anti-metastatic and advanced cancer therapy. RIPK3 may act as a tumor suppressor to inhibit malignant progression of malignant mesothelioma through induction of necrotic apoptosis, whereas RIPK3 DNA epigenetic silencing of methylation impairs necroptosis and leads to chemoresistance as well as poorer prognosis in malignant mesothelioma (115). Tsc1/mTOR has a critical role in suppressing RIPK3 expression and activation in intestinal epithelial cells through TRIM11-mediated ubiquitination and autophagy-dependent degradation. mTOR can act on RIPK3 to enhance the expression and activation of RIPK3 by TNF and microbial pathogen-associated molecular pattern (PAMP)–induced necroptosis. mTOR/RIPK3/necroptosis axis is a driver of intestinal inflammation and cancer (116).

Various components of the scorch pathway are associated with tumorigenesis, invasion, and metastasis, and studies on scorch death have opened up new frontiers in tumor therapy. maternal embryonic leucine zipper kinase (MELK) expression is elevated in lung adenocarcinoma (LUAD) and promotes the malignant progression of LUAD cells by regulating the PLK1-CDC25C-CDK1 signaling pathway to promote proliferation and inhibit apoptosis-mediated cell scorch (16, 117). GSDME-mediated cell scorch death promotes colorectal cancer progression by releasing HMGB1, which induces tumor cell proliferation and PCNA expression through the extracellular regulated protein kinases 1/2 (ERK1/2) pathway (118). Circneil3, a circulating RNA, can act as a sponge by directly binding to mir-1184 and thereby releasing the inhibitory effect of miR-1184. The inhibition of PIF1 by mir-1184 ultimately induces DNA damage and triggers AIM2 inflammasome activation-mediated cell scorching (119). Mediating the cell scorch-induced circneil3/mir-1184/PIF1 regulatory axis may be a promising clinical therapeutic strategy for lung cancer.

Accumulating evidence has identified the crosstalk between ferroptosis, necroptosis, and pyroptosis. A number of factors including NEK7, Tom20, and caspase-1 have been found to mediate the crosstalk between these programmed cell death pathways. For instance, necroptosis and pyroptosis are both able to promote cell lysis. Z-DNA binding protein 1 (ZBP1) has been found to promote pyroptosis and necroptosis upon sensing infection with fungus (120). A well-established cell marker for apoptosis, Bcl-2, is found to regulate pyroptosis and necroptosis by targeting BH3-like domains in GSDMD and MLKL (121). Moreover, caspase-8 plays a key role in switching from necroptosis topyroptosis (122). Moreover, the key molecular regulator for ferroptosis, iron, could promote excessive reactive oxygen species (ROS) production and mediate crosstalk between ferroptosis and necroptosis (123). In the model of myocardial fibrosis, it has been reported that mixed-lineage kinase 3 (MLK3) modulates pyroptosis and ferroptosis *via* distant signaling pathways (124). Interestingly, non-coding RNAs have also been reported to play a key role in the crosstalk between necroptosis, ferroptosis, and pyroptosis (125). All these pieces of evidence highlight the correlation between necroptosis, pyroptosis, and ferroptosis.

## Roles of cell deaths: ferroptosis, necroptosis, and pyroptosis in OC

Apoptosis used to be considered the predominant means of programmed cell death in tumor cells that decide the proliferation rate of cells. Lately, mounting evidence has showed that other types of programmed cell death including ferroptosis, necroptosis, and pyroptosis are highly involved in a variety of cell processes of OC cells, such as chemoresistance and immune response.

### Role of ferroptosis in OC

In brief, the regulatory role of ferroptosis related genes in OC progression was displayed in **Table 1**. Studies have shown that elevated intracellular iron levels are closely associated with OC. FPN was decreased, TFR1 and TF were increased, and iron levels were elevated in high-grade plasma cytotic OC tissues compared with normal ovarian tissues (141). Genetic models of OC initiating cells also exhibit reduced iron efflux pumps and upregulated expression of iron transport–related proteins. This suggests that intracellular iron levels are elevated in OC cells early in the development of OC. Li et al. (126) found that ferrous ammonium citrate (FAC) promoted intracellular iron expression levels in OC cells and inhibited OC cell proliferation, induced apoptosis, promoted inflammatory responses, and inhibited the reduction of lipid peroxides. Inhibition of GPX4 modulated intracellular iron homeostasis and lipid peroxide reduction, induced ferroptosis, and exerted anti-cancer effects.

**Table 1:** The regulatory role of ferroptosis related genes in ovarian cancer progression.

Target	Mechanism	Function	Reference
FAC	Target Fe2^+^ and GPX4	Induce ferroptosis and inhibit ovarian cancer	(126)
SCD1	Target unsaturated fatty acyl chain	Induce ferroptosis and inhibit ovarian cancer	(127)
miR-424-5p	Target ACSL4/erastin/RSL3	Induce ferroptosis and inhibit ovarian cancer	(128)
SPIO-serum	Target GPX4/xCT	Induce ferroptosis and inhibit ovarian cancer	(129)
PARP	Target SLC7A11	Induce ferroptosis and inhibit ovarian cancer	(130)
SNAI2	Target SLC7A11	Induce ferroptosis and inhibit ovarian cancer	(131)
ADAMTS9-AS1	Target miR-587/SLC7A11	Inhibit ferroptosis and promote ovarian cancer	(132)
Sodium molybdate	Target NO/GSH	Induce ferroptosis and inhibit ovarian cancer	(133)
TAZ	Target ANGPTL4/NOX2	Induce ferroptosis and reduce drug resistance	(134)
CBS	/	Inhibit ferroptosis and promote ovarian cancer	(135)
FZD7	Target Tp63	Induce ferroptosis and reduce platinum resistance	(136)
Erastin	Target ROS	Induce ferroptosis and inhibit ovarian cancer	(137)
GALNT14	Target EGFR/mTOR	Induce ferroptosis and reduce platinum resistance	(138)
MAP30	Target Ca2+	Induce ferroptosis and reduce platinum resistance	(139)
Lidocaine	Target miR-382-5p/SLC7A11	Induce ferroptosis and inhibit ovarian cancer	(140)

In ferroptosis, lipid peroxidation driven by ROS plays an important role in the ferroptosis pathway. Tesfay et al. (127) found that steroyl coa desaturase (SCD1) was highly expressed in OC tissues, cell lines, and genetic models of OC stem cells. Inhibition of SCD1 significantly reduced unsaturated fatty acyl chains and increased long-chain saturated ceramides in membrane phospholipids and enhanced the anti-tumor effects of ferroptosis inducers in OC cell lines and *in situ* xenograft models in mice.

OC is associated with abnormal expression of many genes. Ma et al. (128) demonstrated that upregulation of mir-424-5p inhibited ACSL4 expression by directly binding to the 3′-untranslated region (UTR) of ACSL4, thereby reducing erastin- and RSL3-induced ferroptosis and ultimately inhibiting the malignant progression of OC. Mutation of the p53 gene alone caused migration of mouse oviductal epithelial cells; when p53 mutation combined with K-ras activation occurred, mouse oviductal epithelial cells were transformed into tumor cells (142). Zhang et al. (129) found that superparamagnetic iron oxide (SPIO)-serum effectively induced lipid peroxidation and produced large amounts of toxic ROS and promoted the down-regulation of GPX4 and xct, leading to iron-dependent oxidative death. These effects could be reversed using the iron chelator DFO and the lipid peroxidation inhibitor Fer-1. In addition, p53 contributed to promote SPIO-serum–induced ferroptosis in OC cells. Inhibition of PARP downregulates the expression of the cystine transporter protein SLC7A11 in a p53-dependent manner, which, in turn, leads to reduced GSH biosynthesis and promotes lipid peroxidation and ferroptosis (130). SNAI2 inhibits the malignant progression of OC by binding to the promoter region of SLC7A11 and thereby inducing the onset of ferroptosis in OC cells (131). Cai et al. (132) found that ADAMTS9-AS1 can inhibit the malignant progression of OC by regulating the mir-587/SLC7A11 axis to attenuate ferroptosis process in OC and promote proliferation, migration, and invasion of OC cells, leading to malignant progression of OC.

Numerous studies have confirmed that elevated intracellular GSH levels and high expression of related metabolic enzymes are closely associated with drug resistance in OC. Mao et al. (133) found that sodium molybdate induced elevated pools of unstable iron in OC cells and induced GSH depletion by mediating nitric oxide (NO) production and further promoted ferroptosis in OC cells. In addition, NO induces mitochondrial damage through inhibition of mitochondrial aconitase activity, ATP production, and mitochondrial membrane potential, leading to apoptosis in OC cells. Yang et al. (134) found that transcriptional coactivator with PDZ-binding motif (TAZ) removed conferred ferroptosis resistance, whereas overexpression of TAZS89A promoted cellular susceptibility to ferroptosis, and lower TAZ levels were an important reason for reduced ferroptosis susceptibility in chemotherapy-resistant relapsed OC TAZ that can promote OC cell sensitivity to ferroptosis by regulating ANGPTL4 and activating NOX2 entry and exit. Liu et al. (143) established erastin-tolerant cell lines and found that the cell lines could still maintain GSH levels, suggesting the existence of other intracellular pathways for cystine synthesis. The assay revealed high expression of CBS, a key enzyme of the transsulfuration pathway, suggesting that the erastin-tolerant cell line provides cystine to the cells through the upregulated transsulfuration pathway. Verschoor et al. (144) treated an OC cell model using an Xc-system inhibitor and a transsulfuration pathway inhibitor and found that intracellular GSH levels were significantly reduced after transsulfuration pathway inhibition, suggesting that the transsulfuration. Chakraborty et al. (135) found that the expression level of CBS in the transsulfuration pathway was elevated in a few OC cell lines and that CBS gene silencing inhibited cell migration and invasion of OC cells.

The mechanism of action of platinum drug is based on the generation of intracellular ROS, eventually leading to cellular damage and death. Wang et al. (136) found that platinum-resistant cells and tumors exhibited Frizzled 7 (FZD7) expression and that knockdown of FZD7 increased platinum sensitivity and delayed tumorigenesis. In contrast, overexpression of FZD7 activated the oncogenic factor Tp63, which promoted upregulation of the metabolic pathway, leading to platinum resistance in OC cells. Chen et al. (137) found that erastin induced ferroptosis and increased ROS levels, thereby enhancing the cytotoxic effects of cisplatin. Erastin synergistically with cisplatin significantly inhibited OC cell growth. polypeptide N-acetylgalactosaminyltransferase (GALNT14) promoted mtor by modifying EGFR. The combination of mtor inhibitor and cisplatin resulted in a cumulative effect on cell death (138). Santos et al. (145) speculated that reversal of drug resistance by interfering with cysteine metabolism was suggested by the results of a study suggesting that selenium-containing salicin could contribute to a reduction in GSH levels and that an inhibitory effect on CBS was inhibited. Designing a nanodrug could be a new strategy to improve OC treatment.

The mechanism of ferroptosis-related drugs remained to be investigated, but they may be a new pathway to improve OC treatment through the ferroptosis pathway. Chan et al. (139) reported that MAP30 induces an increase in intracellular Ca^2+^ ion concentration, which triggers ROS-mediated cancer cell death through apoptosis and ferroptosis. Natural MAP30 may be used as a non-toxic supplement to enhance chemotherapy in patients with OC with peritoneal metastases. Sun et al. (140) found that lidocaine could inhibit the ferroptosis process in OC cells by enhancing the expression of mir-382-5p in cells, which, in turn, inhibited the ferroptosis process in OC cells by targeting SLC7A11. Lidocaine treatment inhibited tumor growth of OC *in vivo*.

In addition, many other authors have explored the expression levels of ferroptosis-related genes and proteins in patients with OC and the correlation with patient prognosis by bioinformatics analysis. The results of all analyses are presented in **Table 2**.

**Table 2:** Ferroptosis related genes in patients with ovarian cancer.

Related Genes	Diagnostic Potential	Prognostic Potential	Reference
LPCAT3, ACSL3, CRYAB, PTGS2, ALOX12, HSBP1, SLC1A5, SLC7A11, and ZEB1	**√**	**√**	(146)
CDKN1B, FAS, FOS, FOXO1, GABARAPL1, HDAC1, NFKB1,	**×**	**√**	(147)
PEX3, PPP1R15A, SIRT2, IFNG, IL24, MTMR14, and RB1			
ALOX12, ACACA, SLC7A11, FTH1, and CD44	**×**	**√**	(148)
DNAJB6, RB1, VIMP/SELENOS, STEAP3, BACH1, and ALOX12	**√**	**√**	(149)
Staurosporine, epothilone B, DMOG, and HG6-64-1	**×**	**√**	(150)
SLC7A11, RB1, GCH1, LPCAT3, PCBP2, ZFP36, STEAP3,	**√**	**√**	(151)
MAPK8, GABARAPL1, IFNG, PHKG2, HSPA5, MAP1LC3C, and ALOX5			
AC138904.1, AP005205.2, AC007114.1, LINC00665,	**×**	**√**	(152)
UBXN10-AS1 AC083880.1, LINC01558, and AL023583.1			
HIC1, LPCAT3, and DUOX1	**√**	**√**	(153)
AC007848.1, AC011445.1, AC093895.1, AC010336.5, AL157871.2,	**×**	**√**	(154)
AP001033.1, AC009403.1, AC068792.1, LINC01857, LINC00239, and AL513550.1			
FMR1, HNRNPC, METTL16, METTL3, and METTL5	**×**	**√**	(155)

### Role of necroptosis in OC

In brief, the regulatory role of necroptosis related genes in OC progression was displayed in **Table 3**. Hahne et al. (156) explored the ability of the PI3K/AKT inhibitor AEZS-126 alone and in combination with rapamycin to selectively target OC cell proliferation and survival *in vitro*. They found by validation that AEZS-126 exhibited anti-cytotoxicity in an *in vitro* model of OC and that the primary mechanism was the regulation of the necroptotic apoptotic process in OC cells. Mccabe et al. (157)found that inhibitor of apoptosis protein (IAP) plus cystein inhibitor (IZ) treatment selectively induced TNFα-dependent death in several anti-apoptotic cell lines and patient xenografts. Qiu et al. (158) found that upregulation of CD40 may be relatively common in low-grade serous carcinomas (lgscs) and that CD40 activation induced RIP1-dependent, necrosis-like cell death in LGSC cells. Dey et al. (159)found that BMI1 in OC was able to participate in the PINK1-PARK2–dependent mitochondrial pathway and induce a novel non-apoptotic, necroptosis-mediated cell death pattern. Necroptosis enhances the phosphorylation of the downstream substrate MLKL by activating the RIPK1-RIPK3 complex. In addition, inhibition of caspase-8 was found to significantly inhibit NF-κB signaling and lead to necrotic cell death by stabilizing RIPK1 expression (160). Blocking NF-κB signaling and depleting cIAP using SMAC mimics could further render these cells susceptible to necroptosis killing. Increasing caspase-8 expression *in vivo* may be an important tool to improve the prognosis of patients with OC.

**Table 3:** The regulatory role of necroptosis related genes in ovarian cancer progression.

Target	Mechanism	Function	Reference
AEZS-126	Target PI3K/AKT	Induce necroptosis and reduce platinum resistance	(156)
IAPs	Target TNF-α	Induce necroptosis and promote ovarian cancer	(157)
CD40L	Target caspase-3	Induce necroptosis and promote ovarian cancer	(158)
BMI1	Target PINK1-PARK2	Induce necroptosis and promote ovarian cancer	(159)
Caspase8	Target NF-κB and RIPK1	Suppress necroptosis and inhibit ovarian cancer	(160)
Luteal-phase progesterone	Target TNF-a/RIPK1/RIPK3/MLKL	Suppress necroptosis and inhibit ovarian cancer	(161)
CNL	Target MLKL	Induce necroptosis and promote ovarian cancer	(162)
DEBIO 1143	Target cIAP1, XIAP, and caspase-9	Induce necroptosis and promote ovarian cancer	(163)
ALDH1Ai	/	Induce necroptosis and promote ovarian cancer	(164)
CuS–MnS2	/	Suppress necroptosis and inhibit ovarian cancer	(165)
Berberine	Target Caspase-3, Caspase-8, RIPK3, and MLKL	Induce necroptosis and promote ovarian cancer	(166)
RIP1	Target ROS	Induce necroptosis and promote ovarian cancer	(167)

In addition, studies have shown that targeting necroptosis may also promote prognosis in patients with OC. Wu et al. found that luteal-phase progesterone (P4) binds to P4 receptors (prs) and *via* the TNF-a/RIPK1/RIPK3/MLKL pathway in the oviductal epithelium of Trp53M^−/−^ mice, and immortalized human p53-deficient bacterial hair epithelium induces necrotic apoptosis (161), which may be a potential mechanism for progesterone to prevent OC onset. MLKL may be a novel pro-necrotic apoptotic target of ceramide in OC models, and knockdown of MLKL with small interfering RNA (siRNA) significantly abrogated ceramide nanoliposomes (CNL)–induced cell death (162). As a SMAC (second mitochondria-derived activator of caspase) mimetic, DEBIO 1143 was able to reverse resistance to carboplatin by targeting cIAP1, XIAP, and caspase-9 and inducing apoptosis or necroptosis depending on the cell line. Chefetz et al. (164) identified two selective inhibitors of the ALDH1A family (ALDH1Ai) and found that they preferentially killed CD133^+^ OC stem cell-like cells (CSC). ALDH1Ai induced mitochondrial uncoupling proteins and reduced oxidative phosphorylation to induce necrotizing CSC death. In addition, they found that ALDH1Ai was highly synergistic with chemotherapy, significantly reducing tumor initiation capacity and increasing tumor eradication *in vivo*. Chen et al. (165) found that excellent tumor ablation could be achieved by combining treatment with cus-mns2 nanoflowers and 808-nm NIR laser. Cusmns2 nanoflowers could be used as a promising multifunctional nanotheranostic agent for MRI and as a photothermal/photodynamic cancer therapy agent through necroptosis. Liu et al. (166) found that berberine (BBR) could significantly inhibit the proliferative capacity of OC cells in a dose- and time-dependent manner. Combined treatment with BBR and DDP significantly promoted the proportion of necrotic apoptosis in OC cells and had a significant effect on OC cell proliferation and induction of G0/G1 cell cycle arrest. Combined treatment with BBR and DDP significantly increased OC cell death through induction of apoptosis and necroptosis, thereby enhancing the anticancer effects of chemotherapeutic agents. In addition, both ROS-mediated apoptosis and necroptosis could be involved in cisplatin-induced cell death. Therefore, RIP1 can act as a tumor suppressor that promotes the anticancer effects of chemotherapeutic agents such as cisplatin.

### Role of pyroptosis in OC

In brief, the regulatory role of pyroptosis-related genes in OC progression was displayed in **Table 4**. The mechanisms of inflammatory cells in the tumor microenvironment are gradually being understood with the advancement of research. Recent studies have shown that the occurrence and development of OC are closely associated with elevated levels of various inflammatory factors. Qiao et al. (169) found that knockdown of caspase-4 or GSDMD in OC cells significantly inhibited the killing activity of a-NETA cells, suggesting that a-NETA may play a biological role by regulating the cell scorching pathway. Zhang et al. (170) found that nobiletin, a prospective food-derived phytochemical derived from citrus fruits, could induce apoptosis and trigger ROS-mediated cell scorch death by regulating autophagy in OC cells, thereby inhibiting the malignant progression of OC. Liang et al. (168) found that serpentine, a derivative of coumarin, significantly inhibited OC cell growth and induced OC cell death by regulating OC cell apoptosis, cell scorching, and autophagic processes, with good therapeutic promise. HOXA transcript at the distal tip (HOTTIP) was able to increase AKT2 expression and inhibit ASK1/JNK signaling through negative regulation of mir-148a-3p, which, in turn, led to OC cell proliferation and NLRP1 inflammasome-mediated cell scorching process, resulting in OC malignant progression (171).

**Table 4:** The regulatory role of pyroptosis-related targets in ovarian cancer progression.

Target	Mechanism	Function	Reference
Caspase-4/GSDMD	Target a-NETA	Inhibit pyroptosis and suppress ovarian cancer	(156)
Nobiletin	Target ROS	Induce pyroptosis and suppress ovarian cancer	(157)
Osthole	/	Induce pyroptosis and suppress ovarian cancer	(168)
HOTTIP	Target miR-148a-3/AKT2-ASK1/JNK	Induce pyroptosis and promote ovarian cancer	(159)

In addition, many other authors have explored the expression levels offerroptosis-related genes and proteins in patients with OC and the correlation with patient prognosis by bioinformatics analysis. The results of all analyses are presented in **Table 5**.

**Table 5:** Pyroptosis-related genes in patients with ovarian cancer.

Related genes	Diagnostic potential	Prognostic potential	Reference
CASP3, CASP6, AIM2, PLCG1, ELANE, PJVK and GSDMA	**×**	**√**	(172)
GSDMD, GSDMC, GSDME, and PJVK	**×**	**×**	(173)
SLC31A2, LYN, CD44, EPB41L3, VSIG4, FCN1, IRF4, and ISG20	**×**	**√**	(174)
AC006001.2, LINC02585, AL136162.1, AC005041.3, AL023583.1, and LINC02881	**×**	**√**	(175)
DICER1-AS1, MIR600HG, AC083880.1, AC109322.1,	**√**	**√**	(176)
AC007991.4, IL6RAS1, AL365361.1, and AC022098.2			

## Conclusion and prospects

Programmed cell death is a hot issue in biological and medical research, and targeting the cell death process is a common approach in tumor therapy. However, current compounds that induce programmed death are only effective against certain tumor cells, and different types of cancers seem to have different sensitivities to programmed death (177–179). Efforts to understand the sensitivity of different tissue tumors to programmed death are important for the practice of clinical application of programmed death in tumor therapy. Regarding how programmed death is precisely induced *in vivo*, the key regulators of programmed death that have been identified provide important therapeutic targets. Currently, the commonly used anti-tumor drugs in clinical practice have disadvantages such as poor selectivity, toxic side effects, and the tendency to develop drug resistance, which seriously limit their efficacy. According to the characteristics of TCM, studying the effect of TCM and its specific components on programmed tumor death, discovering effective anti-tumor components, and combining multi-disciplinary and multi-disciplinary approaches to target tumor sites by loading programmed death inducers, reactants of programmed death process, or TCM preparations through nanotechnology, so that drug concentrations accumulate at tumor sites, may bring new options for cancer treatment based on programmed death (178, 180, 181). The exploration of Chinese medicine to intervene in programmed death is still in its infancy, but there are already studies, showing extraordinary research prospects by loading Chinese medicine with nanotechnology for tumor treatment (182).

Ferroptosis has become a hot research topic in tumor; the pathway network between iron metabolism, Fenton reaction, Xc-system, and GPX4 has been initially established (183, 184); and other important related pathways (including transsulfur pathway) and ferroptosis-related drugs need to be further investigated. Current chemotherapy regimens for OC are still dominated by platinum and paclitaxel drugs, but the prognosis of patients with advanced OC remains bleak. An in-depth investigation of the link between ferroptosis pathway and OC will facilitate the search for a new chemotherapeutic regimen. Ferroptosis inducers and inhibitors are expected to be used effectively and rationally in the future, thus improving the precision of treatment for OC and other tumors. As a newly discovered mode of programmed cell death, necroptosis is closely related to a variety of case-physiological processes (185, 186), and most of the studies related to necroptosis are at the stage of basic experiments. Necroptosis plays an opposing role in anti-tumor; on the one hand, it can inhibit the proliferation and migration of tumor cells; on the other hand, it can play a pro-tumor growth role and participate in early tumor formation. Further in-depth study on the molecular mechanism of necroptosis pathway and the relationship between upstream and downstream signaling molecules of related signaling pathways, to explore its role in OC in different modes and to find corresponding target drugs, is one of the future directions to improve the therapeutic effect of OC. Cell scorch is a programmed cell death mediated mainly by inflammatory caspases (177). NLRP3 inflammatory vesicles activate caspase-1, which, in turn, causes pro–IL-1β and pro–IL-18 to form mature IL-1β and IL-18 and trigger cellular inflammation (187, 188). At the same time, caspase-1 cleaves the downstream factor abscisicin D. These actions create active pores in the cell membrane and lead to the onset of cellular scorching. The occurrence and development of OC are closely related to the inflammatory response, so the cell scorching caused by inflammatory vesicles/factors may play an important role in OC. A variety of Chinese herbal components and formulations have regulatory effects on cell scorch, and with further research, Chinese medicine may be used to regulate cell scorch in the prevention and treatment of OC.

In conclusion, this article reviews the progress of research on ferroptosis, necroptosis, or pyroptosis in the development of OC and after prognosis and treatment. Nevertheless, the exact roles of ferroptosis, necroptosis, and pyroptosis in OC remain to be fully elucidated. It is important to investigate the molecular mechanisms and physiopathological roles of ferroptosis, necroptosis, and pyroptosis and to specifically design the corresponding drug therapy for OC.

## Author Contributions

Original draft preparation, allocation, supplementation, and editing: CZ. Revision: NL and CZ. All authors have read and agreed to the published version of the manuscript.

## Funding

This work was supported by the internal fund of Shenjing Hospital of China Medical University (Grant No. M0797).

## Conflict of Interest

The authors declare that the research was conducted in the absence of any commercial or financial relationships that could be construed as a potential conflict of interest.

## Publisher’s Note

All claims expressed in this article are solely those of the authors and do not necessarily represent those of their affiliated organizations, or those of the publisher, the editors and the reviewers. Any product that may be evaluated in this article, or claim that may be made by its manufacturer, is not guaranteed or endorsed by the publisher.

## Abbreviations

OC, ovarian cancer; RCD, regulated cell death; ACD, accidental cell death; TLRs, Toll-like receptors; GSDMB, Gasdermin B; ACSL4, acyl-coa synthetase long-chain family member 4; LPCAT3, lysophosphatidylcholine acyltransferase 3; LOX, lipoxygenase; NFS1, nitrogen fixation 1; TFRC, transferrin receptor; FTH1, ferritin heavy chain 1; NCOA4, nuclear receptor coactivator 4; NADH, nicotinamide adenine dinucleotide; RIPK1, serine/threonine protein kinase 1; MLKL, mixed lineage kinase domain–like pseudokinase; caspase-1, cysteine-aspartic proteases 1; FADD, Fas-associating protein with a novel death domain; TNF-α, tumor necrosis factor α; TRADD, TNFR1-associated death domain protein; LU-BAC, linear ubiquitin chain assembly complex; NF-κB, nuclear factor κB; MAPK, mitogen-activated protein kinase; RHIM, RIP homotypic interaction motif; PRRs, pattern recognition receptors; ASC, apoptosis-associated speck-like protein; NLRP1, nucleotide-binding oligomerization domain–like receptor protein 1; PYD, pyrin domain; CARD, caspase activation and recruitment domain; TAMs, tumor-associated macrophages; GPX4, glutathione peroxide 4; PAMP, pathogen-associated molecular pattern; LUAD, lung adenocarcinoma; FAC, ferrous ammonium citrate; FZD7, Frizzled 7; IAP, inhibitor of apoptosis protein; LGSCs, low-grade serous carcinomas; CNL, ceramide nanoliposomes; BBR, berberine.
